# Nanoencapsulation of Organic Phase Change Materials in Poly(3,4-Ethylenedioxythiophene) for Energy Storage and Conversion

**DOI:** 10.3390/polym16010100

**Published:** 2023-12-28

**Authors:** Inés Adam-Cervera, Jose Huerta-Recasens, Clara M. Gómez, Mario Culebras, Rafael Muñoz-Espí

**Affiliations:** Institute of Materials Science (ICMUV), University of Valencia, Catedràtic José Beltrán 2, 46980 Paterna, Spain; ines.adam@uv.es (I.A.-C.); jose.huerta@uv.es (J.H.-R.); clara.gomez@uv.es (C.M.G.)

**Keywords:** phase change material (PCM), thermoelectric generator (TEG), conducting polymer, miniemulsion polymerization, nanoencapsulation, PEDOT, oxidative polymerization, latent heat storage

## Abstract

This work focuses on the encapsulation of two organic phase change materials (PCMs), hexadecane and octadecane, through the formation of nanocapsules of the conducting polymer poly(3,4-ethylenedioxythiophene) (PEDOT) obtained by oxidative polymerization in miniemulsion. The energy storage capacity of nanoparticles is studied by preparing polymer films on supporting substrates. The results indicate that the prepared systems can store and later release thermal energy in the form of latent heat efficiently, which is of vital importance to increase the efficiency of future thermoelectric devices.

## 1. Introduction

Current global issues of energy shortage and environmental deterioration have sparked an enormous interest in the research and development of systems capable of collecting and generating energy in a more efficient and sustainable way. A system that has gained particular attention in this context is the thermoelectric generator (TEG) [1].

A TEG is a solid-state energy conversion device that can convert low-grade heat into electricity directly by using thermoelectric materials based on the Seebeck effect [2]. Advantages of TEGs, such as compact manufacturing, no fluid leakages, robustness and reliability, and low maintenance costs [3] represent an enormous potential for their use in many applications, including aerospace engineering [4], solar power generation [5], waste heat utilization [6], medicine [7], electronic devices [8], the automotive industry [9], and ocean power generation [10]. The thermoelectric efficiency of a material is given by the dimensionless figure of merit, *ZT* = *S*^2^ *σ T*⁄*κ*, where *T*, *S*, *σ*, and *κ* are the absolute temperature, Seebeck coefficient, electrical conductivity, and thermal conductivity, respectively. Thermoelectric materials can be classified into inorganic and organic semiconductors. Although inorganic semiconductors are the materials with the highest *ZT* values [11], several issues related to material equipment, such as low cost, lightweight, and flexibility required in portable thermoelectric generators, have promoted the use of organic materials with low thermal conductivity, high flexibility, and good processability. Organic semiconductors (e.g., conducting polymers and carbonaceous materials) have been proposed as possible candidates due to their availability, low thermal conductivity, ease of chemical modification, and scale-up potential [12,13,14].

In the case of conductive polymers, there has been a notable advancement in their thermoelectric performance, mainly as a result of effective doping mechanisms in PEDOT, which have enhanced electrical conductivity, leading to *ZT* values of ~0.2–0.4 [15]. Consequently, this development places PEDOT as one of the most promising conductive polymers for thermoelectric applications. From an application perspective, TEGs should be subjected to a large and stable temperature difference between hot and cold ends to enhance their performance and maximize the power output. However, TEGs are usually working under transient or periodic operating conditions, mainly determined by the heat sources. These sources often cannot provide a constant heat supply, and there is a need to ensure that all the heat is dissipated from the cold end to prevent thermal equilibration between the hot and cold ends of the TEG. In this sense, several possible solutions have been proposed, including the utilization of both passive and active cooling methods to manage heat dissipation from the cold end of the TEG, although these techniques have been found to be inefficient. Another approach involves the use of materials with the capability to store heat and subsequently release it, enabling more effective utilization in TEG systems. These materials can be the so-called phase change materials (PCMs), which are characterized by having the inherent ability to absorb, store, and release a large amount of energy in the form of latent heat while a transition between two phases occurs. PCMs have been studied for many applications, such as thermal storage [16,17], thermal switches [18], thermal energy storage in buildings and construction [19], thermoregulation in textiles [20], thermal barriers [21], food packaging [22], photo-responsive materials [23], and thermotherapy [24]. In recent years, they have also been applied in the context of thermoelectric materials [25,26].

Depending on the phase transition, PCMs can be classified as solid–solid, solid–liquid, solid–gas, or liquid–gas PCM. Among these types, solid–liquid transition materials are particularly noteworthy due to their extensive applicability, mainly owing to their broad working temperature range, high latent heat of melting, and the capability to undergo a manageable change in volume [27]. In turn, solid–liquid PCM can be divided into organic and inorganic. Organic materials are characterized by high latent heat, congruent fusion, and high thermal stability with small volume changes but have low thermal conductivity. Inorganic materials, which have a very high latent heat and a moderate thermal conductivity, have, unfortunately, several handicaps: they can be corrosive, undergo undercooling, and most also undergo phase segregation and large volume changes.

Considering the advantages and limitations of the different types of materials, organic PCMs are probably the ones with the greatest potential to be used in TEG-based organic materials. However, to use organic PCMs effectively, it is necessary to overcome several limitations related to processability, reuse, and cyclability of the thermal process, as well as leakage to the environment. In this sense, one of the most common strategies is the encapsulation of PCM in micrometric and nanometric capsules [28,29]. The encapsulation provides a stable confinement that prevents the PCM from coming into contact with the surrounding environment. This approach ensures the protection of the phase change material (PCM) within the capsule because it guarantees its stability over multiple heat absorption and release cycles, maintains a relatively constant volume during the phase transition, and significantly increases the surface area, thereby increasing as well the heat transition area and improving the thermal conductivity of the system [29,30,31,32].

An efficient and versatile technique for the nanoencapsulation of PCMs has been miniemulsion polymerization [29,33,34]. Polymeric shells are stable in a reasonable temperature range, hermetic, and flexible, which allows them to adapt perfectly to changes in the volume of the PCM. To incorporate PCMs into thermoelectric materials effectively, the capsules should be conductive, which is crucial to minimize any adverse effects on the overall thermoelectric performance. Therefore, we report here the use of a conductive polymer, namely PEDOT [35,36], as a shell material for the effective encapsulation of organic PCMs. 

Regarding the integration of PCMs in TEG systems, most of the studies conducted so far consider the PCM as an external component of the thermoelectric generator, which results in a modest enhancement of the final thermoelectric performance [37,38,39,40,41,42,43,44,45,46,47]. No studies have reported the incorporation of PCMs within the thermoelectric material, which can potentially improve the heat transport and performance of the final device. In this work, we present the use of nanoencapsulation of the PCM as a strategy to achieve an intimate integration with the actual thermoelectric material. Specifically, we encapsulate two organic phase change materials, hexadecane and octadecane, through the formation of nanoparticles of the conductive polymer PEDOT by oxidative polymerization in miniemulsion. After developing the methodology and characterizing the materials, the thermal energy storage capacity of nanoparticles is evaluated by preparing polymer films on supporting substrates.

## 2. Materials and Methods

### 2.1. Materials

Octadecane (99.9%), hexadecane (99%), iron(III) *p*-toluenesulfonate hexahydrate (99.9%), hydrogen peroxide solution (30 wt.% in H_2_O), and poly(diallyldimethylammonium chloride) solutions (average *M*_w_ < 100,000, 35 wt.% in H_2_O; average *M*_w_ = 200,000–350,000, 20 wt.% in H_2_O; and average *M*_w_ = 400,000–500,000, 20 wt.% in H_2_O) were purchased from Sigma-Aldrich/Merck (Darmstadt, Germany); 3,4-ethylenedioxythiophene (EDOT, 99%) was acquired from Alfa Aesar (Karlsruhe, Germany). All chemicals were used as received without further purification. Ultrapure MilliQ water was used for all the experiments.

### 2.2. Preparation of PEDOT/PCM Nanoparticles

The preparation of PEDOT nanoparticles was carried out by oxidative polymerization in miniemulsion, using iron(III) *p*-toluenesulfonate hexahydrate as oxidant and poly(diallyldimethylammonium chloride) (PDADMAC). On the one hand, the aqueous phase consisted of 40 mL of 0.5 wt.% PDADMAC aqueous solution, prepared by diluting the commercial concentrated solution. On the other hand, the organic phase was prepared by mixing EDOT (0.200 mL) and hexadecane or octadecane (0.100 or 0.150 mL, depending on the EDOT/PCM ratio used). Both phases were stirred separately for 5 min at 850 rpm in a 35 °C bath, subsequently mixed, pre-emulsified under stirring for 45 min (850 rpm, 35 °C bath), and emulsified by ultrasonication in a Branson SFX 550 digital sonifier (Branson Ultrasonics, Brookfield, CT, USA; 1/2″ tip, 70% amplitude, 1.0/0.1 s pulse/pause sequence intensity, 6 min). The oxidative polymerization started with the addition of a solution of iron(III) *p*-toluenesulfonate hexahydrate (1.903 g) in water (10 mL) by dosing with a Harvard 33 syringe pump (1 h, 10 mL·h^−1^) under magnetic stirring (850 rpm, 35 °C bath). Three hours later, hydrogen peroxide (50 µL of a 30 wt.% aqueous solution) was added, and the system was further left to react for 16 h. For reference, samples without PCM were prepared analogously but without hexadecane or octadecane.

### 2.3. Preparation of PEDOT/PCM Films on Supporting Substrates

For the evaluation of the thermal energy storage of the materials, PEDOT and PEDOT/PCM hybrid films were prepared by drop-casting a dispersion of PEDOT nanocapsules with and without PCM on copper substrates. Water was subsequently evaporated at room temperature in a fume hood. For the electric conductivity measurements, analogous films were prepared on poorly conductive glass substrates. All experiments were reproduced three times.

### 2.4. Characterization Techniques

The size of the PEDOT nanoparticles was determined by dynamic light scattering (DLS) with a Zetasizer Nano ZS equipment (Malvern Instruments, Madrid, Spain). Transmission electron microscopy (TEM) was carried out in a JEOL JEM 1010 microscope (Tokyo, Japan) equipped with a digital camera AMT RX80 and working at an acceleration voltage of 100 kV. Scanning electron microscopy (SEM) was conducted in a Hitachi SU-8000 scanning electron microscope (Tokyo, Japan), operated at different voltages under deceleration mode.

The thermal behavior of capsules (after evaporation of water) was evaluated by differential scanning calorimetry (DSC) using DSC Q20 TA equipment (TA Instruments, New Castle, DE, USA). The samples were submitted to a thermal treatment of a minimum of 10 cycles of heating and cooling (from −15 °C up to 45 °C, at 5 °C min^−1^). Thermogravimetric analysis (TGA) was carried out with a TGA 550 TA thermobalance (TA Instruments). The samples were heated from room temperature to 800 °C in two heating ramps: from RT °C to 50 °C, at 40 °C min^−1^, and from 50 °C to 800 °C, at 10 °C min^−1^.

The thickness of the prepared films was measured using an Alpha-Step D-500 profilometer (KLA, Milpitas, CA, USA). The values obtained were (410 ± 30) nm for the PEDOT/hexadecane sample, (385 ± 20) nm for the PEDOT/octadecane, and (510 ± 13) nm for the film with PEDOT nanoparticles without PCM.

The electrical conductivity of the synthesized samples was determined by a four-point probe method on glass substrates using a Signatone manual sheet resistivity measurement system, where the sheet resistance of the prepared films (30 × 30 mm) was measured between tips and multiplied by the film thickness. 

The Seebeck coefficient was determined as the ratio between the electrical potential (Δ*V*), measured with an Agilent 34401 digital multimeter (Agilent Technologies, Santa Clara, CA, USA), and the temperature difference (Δ*T*), measured using PT100 resistors connected to a Lakeshore 340 temperature controller.

## 3. Results and Discussion

### 3.1. PEDOT Nanoparticles without and with PCMs

The preparation of PEDOT nanoparticles was performed by oxidative polymerization in miniemulsion, using iron(III) *p*-toluenesulfonate hexahydrate as an oxidant and poly(diallyldimethylammonium chloride) (PDADMAC) as a stabilizer (see reaction scheme in Scheme S1, Supporting Information). In initial trials, we tested the effect of the PDADMAC molecular weight on the resulting morphology of the particles. For this purpose, solutions of low (average *M*_w_ ≤ 100,000), medium (average *M*_w_ = 200,000–350,000), and high (average *M*_w_ = 400,000–500,000) molecular weights were used. PEDOT nanoparticles prepared with low and high molecular weights PDADMAC showed a strong tendency to form aggregates, as evaluated from SEM images (Figure S1, Supporting Information). Low molecular weight PDADMAC does not sufficiently stabilize the EDOT droplets, so particles of different sizes and aggregates are formed. When high molecular weight PDADMAC is used, the presence of long polymer chains at the monomer droplet interface appears to slow down the polymerization and prevent part of EDOT from being oxidized, which causes incomplete polymerization that leads to the formation of a mixture of capsules and particles of non-uniform morphology and size. In contrast, the most uniform particles were obtained with the medium molecular weight, and this was the one used in the rest of the experiments (Figure S2, Supporting Information).

Encapsulating phase change materials within polymer nanoparticles offers an effective means to ensure the stability and cyclability of heat storage and release systems. This approach leverages the inherent ability of the polymer particles to withstand the pressure–volume changes experienced by the PCMs. However, the insulating properties of most polymers decrease the efficiency of heat transfer between the PCM and the surroundings. Consequently, as previously mentioned, the present study aims to enhance this heat transfer by encapsulating the PCMs within conductive polymer capsules, specifically utilizing PEDOT, which facilitates improved thermal conduction between the systems.

Hexadecane and octadecane alkanes were chosen as phase change materials as they possess large melting enthalpies and melting temperatures between 18 and 30 °C, which make them promising candidates for applications requiring thermal energy storage near room temperature. In addition, these compounds are commonly used as osmotic pressure agents to stabilize miniemulsion systems, so they are especially convenient regarding the stabilization of the colloidal system during the formation of the polymer capsules.

**Figure 1:**
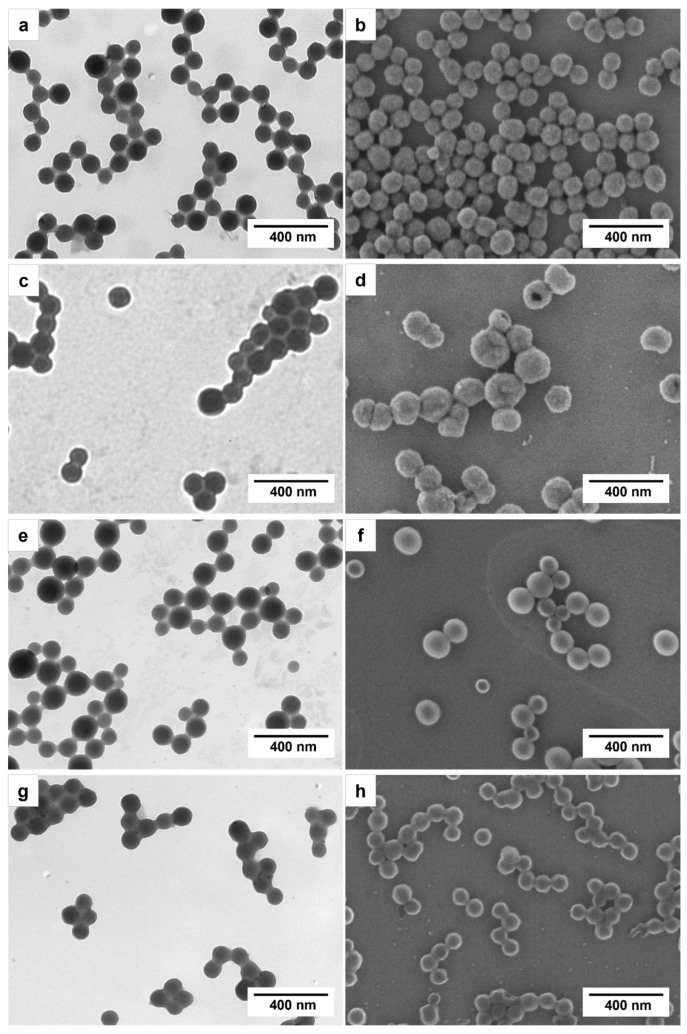
TEM (**left**) and SEM (**right**) images of PEDOT nanoparticles prepared at the following monomer–PCM ratios: (**a**,**b**) EDOT–hexadecane 2:1, (**c**,**d**) EDOT–octadecane 2:1, (**e**,**f**) EDOT–hexadecane 4:3, (**g**,**h**) EDOT–octadecane 4:3.

The encapsulation of the PCMs was carried out with two different EDOT–alkane ratios: 2:1 and 4:3 (*v*/*v*). The prepared particles showed a spherical morphology and a relatively uniform size, as observed in the TEM and SEM images presented in Figure 1.

Thermogravimetric analysis (TGA) (traces shown in Figure S3, Supporting Information) did not show, as expected, a differentiated mass loss for the alkane. This may be attributable to a very intimate incorporation of the alkane in the PEDOT matrix, forming nanometric domains within the structure. In addition, a considerable amount of PDADMAC may remain integrated into the final material even after several washing cycles, so it becomes difficult to distinguish the steps in the TGA curve. The prepared PEDOT/PCM particles were also studied by X-ray diffraction (patterns presented in Figure S4, Supporting Information). Crystalline reflections were observed for both PEDOT/PCM systems, PEDOT/hexadecane, and PEDOT/octadecane. Considering that the prepared PEDOT is amorphous and the reflections do not correspond to the oxidant agent, iron(III) *p*-toluenesulfonate (also measured as a reference), the results suggest the presence of the PCMs in the samples. However, the analysis is complex because the formation of ordered structures between the remaining *p*-toluenesulfonate and the structuring agent (PDADMAC) cannot be excluded. Therefore, any quantification based on XRD results is very challenging.

**Figure 2:**
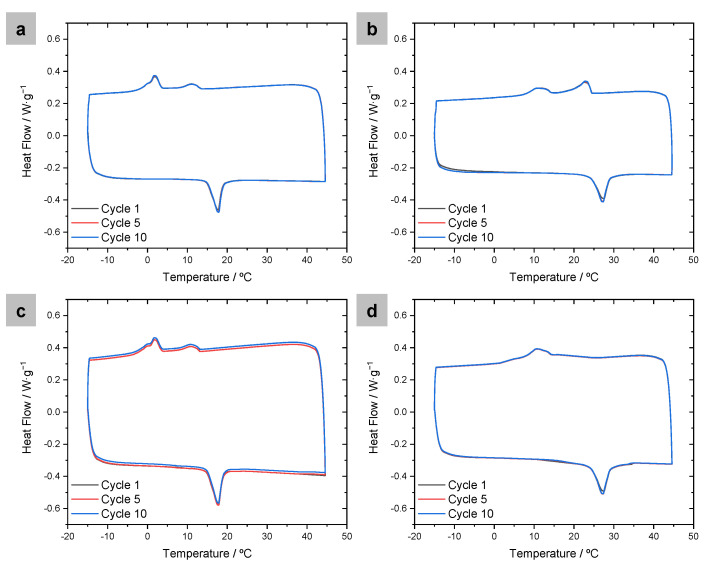
DSC thermogram of PEDOT nanoparticles prepared at the following monomer–PCM ratios: (**a**) EDOT–hexadecane 2:1, (**b**) EDOT–octadecane 2:1, (**c**) EDOT–hexadecane 4:3, (**d**) EDOT–octadecane 4:3.

The thermal storage capacity of the prepared capsules was studied by differential scanning calorimetry (DSC), subjecting the materials to ten heating and cooling cycles between −15 and 45 °C. Figure 2 shows the DSC traces of the four prepared systems. PEDOT nanoparticles with no PCM were also measured as a reference, and no crystallization or melting points were observed (see Figure S5, Supporting Information). DSC traces for all PEDOT/PCM samples present an endothermic melting peak in the heating cycles and an exothermic crystallization peak while cooling. The energy storage capacity of each system was calculated by integrating the areas of the corresponding melting peaks. Considering that the melting heat flow should result from the PCM contained in the sample and knowing the initial amount of PCM introduced in the formulation, we estimated the encapsulation efficiency of the PCM. The corresponding results are shown in Table 1. While the absolute values of melting enthalpies obtained are low, they are reasonable for the amount of PCM introduced in the initial systems, with encapsulation efficiencies close to 50% for the samples with EDOT/PCM ratio 4:3 and close to 70% for the samples with ratio 2:1.

### 3.2. PEDOT/PCM Coatings on Supporting Substrates

**Figure 3:**
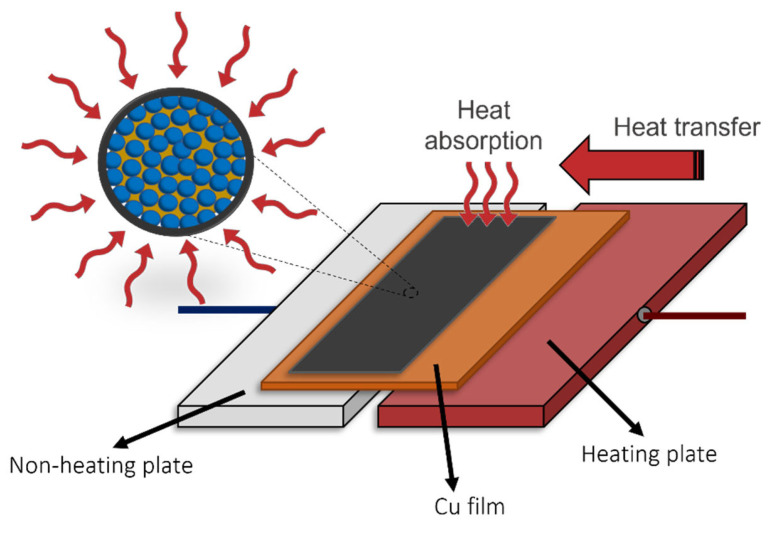
Representation of the heat transfer measurement system on a PEDOT/PCM-coated copper substrate.

The thermal storage capacity of the nanoparticles was assessed by comparing the temperature difference between a substrate containing PCM and one without, both subjected simultaneously to the same amount of heat. The substrate was prepared by coating three layers of the nanoparticles (with and without the encapsulated alkane) on copper substrates. The system used for the measurements (Figure S6, Supporting Information) consisted of two plates (a heating plate and a non-heating plate), the prepared Cu film, and two Pt100 temperature sensors, one placed on the side of the Cu film that contains the nanoparticle suspension layer (S_1_), and the other one on the side without coating (S_2_). Thermal paste was used to favor the efficiency of heat transmission between the plates and the prepared polymer-coated copper substrate. Note that the heating plate is only in contact with the part of the Cu film that does not contain nanoparticles, and the non-heating plate is only in contact with the part of the film that contains nanoparticles. This approach creates a heat flux moving from the region lacking nanoparticles towards the region where the nanoparticles are situated, as illustrated in Figure 3.

The measurements involved applying consistent heating power to the heating plate (voltage applied to the Peltier plate). This method allowed for the controlled, gradual increase in the temperature of the substrate until the predetermined temperature was reached. It was decided to set the designated temperature when the thermocouple, positioned on the surface of the Cu film containing the layers of the nanoparticle suspension, reached 34 °C, ensuring that the PCM had completed the phase transition. At that moment, the potential difference was no longer applied, and the substrate was allowed to cool independently. The entire process was monitored using the two Pt100 thermocouples that record temperature as a function of time. The described measurement process was conducted three times for each sample.

**Figure 4:**
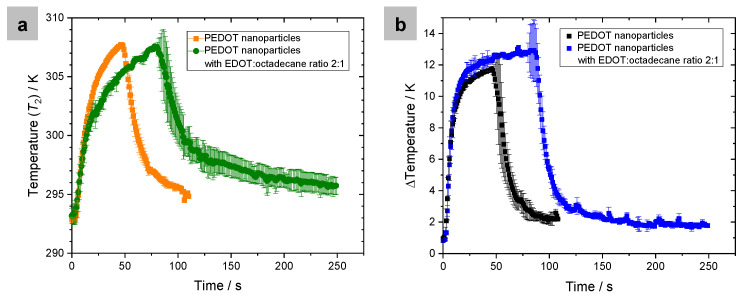
Graphic representation of the temperatures recorded in a system with PEDOT nanoparticles without PCM and a system with octadecane-containing nanoparticles (EDOT–octadecane ratio of 2:1). (**a**) Temperature recorded in the thermocouple in contact with the surface of the Cu film containing the nanoparticles (*T*_2_) as a function of time. (**b**) Temperature difference between the thermocouples S_1_ and S_2_ for each system. Error bars in the graphs result from the standard deviation of averaging three measurements for each sample.

For illustration purposes, Figure 4 displays the graphical representation of the results obtained from the measurements conducted on a system with PEDOT nanoparticles without PCM and a system with octadecane-containing nanoparticles (EDOT–octadecane ratio of 2:1). Figure 4a represents the temperature as a function of time recorded in the thermocouple in contact with the surface of the Cu film containing the nanoparticles (*T*_2_), and Figure 4b represents the Δ*T* calculated from the difference between the temperature registered in the thermocouple S_1_ and S_2_ for each system. The orange and green curves in the first graph correspond to the temperature recorded in the thermocouple in contact with the nanoparticles (S_2_), and the black and blue curves of the second graph correspond to the Δ*T* calculated from the difference between temperatures recorded by thermocouples S_1_ and S_2_. Figure 4a clearly illustrates that the thermocouple of the system that contains the nanoparticles without PCM (orange curve) quickly reaches the maximum established temperature (*T*_2_ = 307 K); when no heat is supplied, the system starts to cool down rapidly. In contrast, the curve corresponding to the temperature recorded by the thermocouple of the system containing the PCM (green line) takes much longer to reach the maximum set temperature, and when the heat supply stops, the cooling down is slow. The difference between the orange and the green curves is caused by the presence of the PCM in the system. As the system containing the PCM is heated up, the PCM absorbs some of the heat, thus causing a delayed increase in sample temperature to reach *T*_2_. Conversely, as the temperature decreases, the PCM begins to release heat, moderating the descent of the curve. The effect of the PCM can also be seen by comparing the black and blue curves in Figure 4b. The temperature difference between thermocouples S_1_ and S_2_ of both systems is the same until the PCM starts to show its effect, with less heat reaching thermocouple S_2_, so that Δ*T* of the system with PCM increases with respect to the Δ*T* of the system without PCM.

Table 2 shows the electrical conductivity, Seebeck coefficient, and power factor obtained for substrates coated with PEDOT nanoparticles without PCM, with octadecane-containing nanoparticles (EDOT–octadecane ratio of 2:1), and with hexadecane-containing nanoparticles (EDOT–hexadecane ratio of 2:1). For experimental reasons, poorly conductive glass substrates were used as support for these measurements. While the encapsulation of the PCM can lead to a decrease in the electrical conductivity of PEDOT due to the incorporation of an insulating material, the observed conductivity range remains comparable to that of low-doped semiconductors. It is crucial to note that in the final device, the entire thermoelectric element will not rely solely on the material containing the PCM. Instead, this material will constitute only one end of the thermoelectric leg. Consequently, the incorporation of PCM capsules is anticipated to have a minimal impact on the overall thermoelectric efficiency of the module.

## 4. Conclusions

This work reports the encapsulation of organic phase change materials (PCM) in poly(3,4-ethylenedioxythiophene) (PEDOT) nanoparticles by miniemulsion oxidative polymerization. Hexadecane and octadecane were chosen as PCMs due to their high melting enthalpy and application temperature in the range of 18 to 35 °C, which makes them promising candidates for applications that require thermal energy storage at temperatures close to room temperature.

The results obtained indicate the effective encapsulation of the two PCMs, revealing that the conductive nanoparticles formed possess a spherical and homogeneous morphology without the presence of aggregates. The applicability of the prepared PEDOT/PCM nanoparticles in coatings was demonstrated by preparing polymer layers on substrates. The thermal studies conducted exhibit not only satisfactory but also reproducible outcomes over time, providing evidence of the robustness and reliability of the developed systems in storing thermal energy through latent heat. 

This study provides insights into the encapsulation of phase change materials within conductive nanoparticles, offering opportunities for further exploration and optimization of these systems specifically for enhancing the efficiency of thermoelectric devices in energy storage and thermal management. 

## Figures and Tables

**Table 1:** Experimental values of the melting temperature (*T*_m_), the melting enthalpy, and calculated encapsulation efficiency from differential scanning calorimetry (DSC) data.

Sample	Encapsulated PCM	*T*_m_/°C	Δ*H*_m_/J·g^−1^	Encapsulation Efficiency/%
EDOT–hexadecane 2:1	Hexadecane	18. 0	5.9	67.1
EDOT–hexadecane 4:3	Hexadecane	17.1	6.4	49.7
EDOT–octadecane 2:1	Octadecane	27.8	6.5	67.7
EDOT–octadecane 4:3	Octadecane	26.3	6.8	48.5

**Table 2:** Values of conductivity, Seebeck coefficient, and power factor (PF) of polymer films with and without PCM deposited on glass substrates.

Sample	Conductivity σ/S·cm^−1^	Seebeck Coefficient/mV·K^−1^	PF/µW·K^−2^ ·m^−1^
Only PEDOT	(1.7 ± 0.2) × 10^−1^	0.022 ± 0.002	(8.2 ± 1.4) × 10^−3^
EDOT–octadecane 2:1	(7.4 ± 0.4) × 10^−4^	0.28 ± 0.05	(5.9 ± 1.5) × 10^−3^
EDOT–hexadecane 2:1	(1.9 ± 0.6) × 10^−2^	0.105 ± 0.007	(2.1 ± 0.2) × 10^−2^

## Data Availability

The data presented in this study are available upon request from the corresponding authors.

## Supplementary Materials

The following supporting information can be downloaded at: https://www.mdpi.com/article/10.3390/polym16010100/s1. Scheme S1: scheme of oxidative polymerization in miniemulsion process of the ethylenedioxythiophene (EDOT); Figure S1: TEM micrographs of PEDOT nanoparticles prepared with poly(diallyldimethylammonium chloride) (PDADMAC) of different molecular weights as a stabilizer; Figure S2: TEM and SEM micrographs of PEDOT particles prepared with PDADMAC as a stabilizer of average molecular weight *M*_w_ = 200,000–350,000; Figure S3: TGA curves of PEDOT nanoparticles prepared without and with PCM; Figure S4: X-ray diffraction patterns of PEDOT/octadecane and PEDOT/hexadecane nanoparticles prepared at EDOT–PCM ratios of 2:1 and 4:3. Octadecane, hexadecane, and iron(III) tosylate are also included as references; Figure S5: DSC curves of PEDOT nanocapsules without PCM; Figure S6: representation of the heat transfer measurement system on the Cu film.
