# Supplementary material for: Nanoencapsulation of Organic Phase Change Materials in Poly(3,4-Ethylenedioxythiophene) for Energy Storage and Conversion
Source: Polymers (Basel). 2023 Dec 28;16(1):100. doi: 10.3390/polym16010100 (PMC10780879; doi:10.3390/polym16010100)
Supplement: Supplementary file 1 [file polymers-16-00100-s001.zip › polymers-2716639-supplementary.pdf]

SUPPORTING INFORMATION

Nanoencapsulation of Organic Phase Change Materials in Poly(3,4-Ethylenedioxythiophene) for Energy Storage and Conversion

Inés Adam-Cervera, Jose Huerta-Recasens, Clara M. Gómez, Mario Culebras * and Rafael Muñoz-Espí *

Institute of Materials Science (ICMUV), University of Valencia, Catedràtic José Beltrán 2, 46980, Paterna, Spain
* Correspondence: mario.culebras@uv.es (M.C.); rafael.munoz@uv.es (R.M.-E.)

Table of Contents

1. Oxidative Polymerization of 3,4-Ethylenedioxythiophene 2

2. Optimization of Nanoparticle Preparation: TEM Micrographs.......................... 2

3. TEM and SEM Micrographs of PEDOT Nanoparticles....................................... 3

4. TGA Curves of PEDOT Nanoparticles with and without PCM 3

5. X-ray Diffraction Patterns.. 4

6. DSC Curves of PEDOT Nanoparticles.. 5

7. System for Heat Transfer Measurement 5

1. Oxidative Polymerization of 3,4-Ethylenedioxythiophene

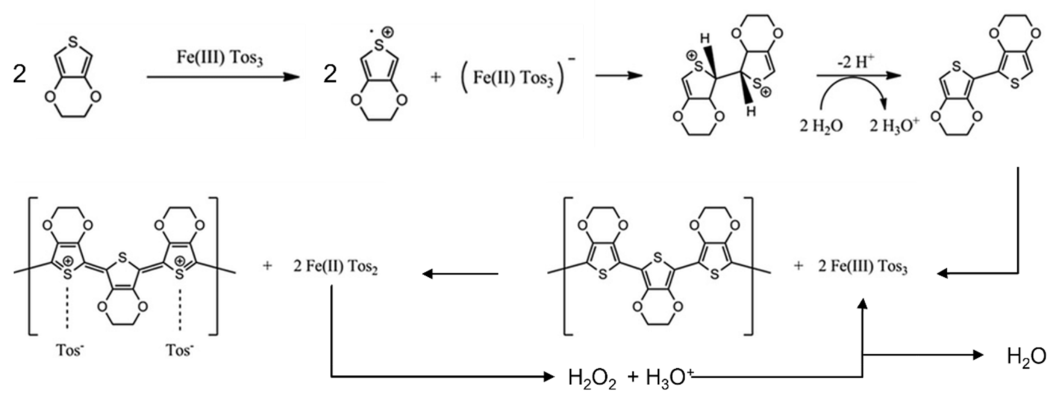

Scheme S1. Scheme of oxidative polymerization of ethylenedioxythiophene (EDOT).

2. Optimization of Nanoparticle Preparation: TEM Micrographs

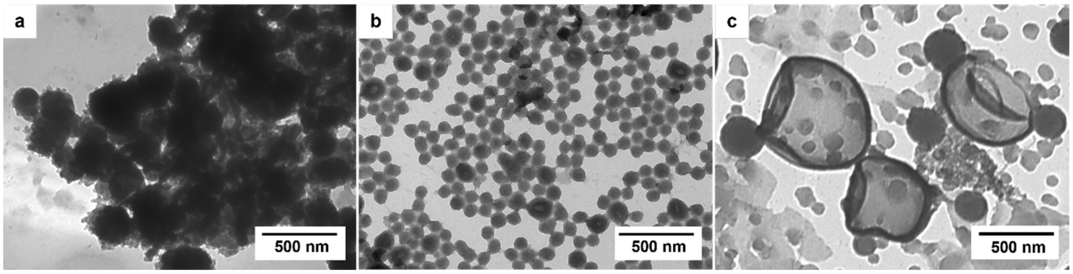

Figure S1. TEM micrographs of PEDOT nanoparticles prepared with poly(diallyldimethylammonium chloride) (PDADMAC) of different molecular weights as a stabilizer: (a) average $M_w < 100,000$, (b) average $M_w = 200,000–350,000$, and (c) $M_w = 500,000$.

3. TEM and SEM Micrographs of PEDOT Nanoparticles

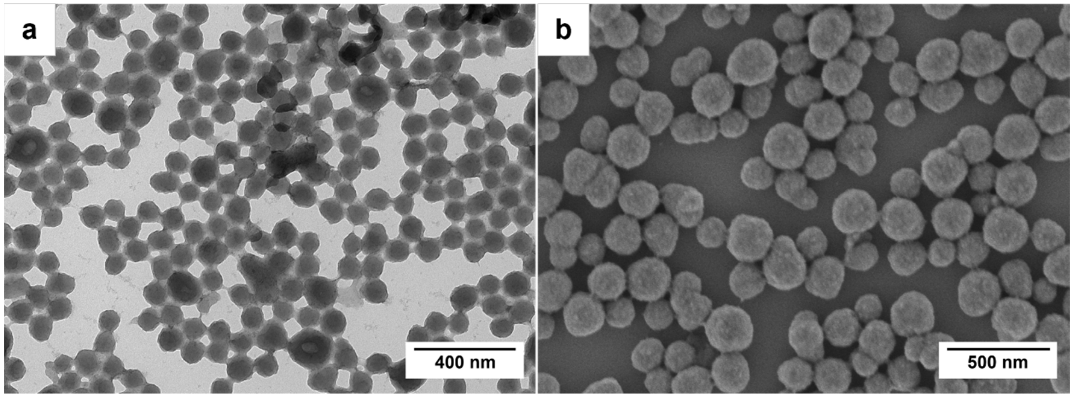

Figure S2. TEM (a) and SEM (b) micrographs of PEDOT particles prepared with PDADMAC as a stabilizer of average molecular weight M_w = 200,000–350,000.

4. TGA Curves of PEDOT Nanoparticles with and without PCM

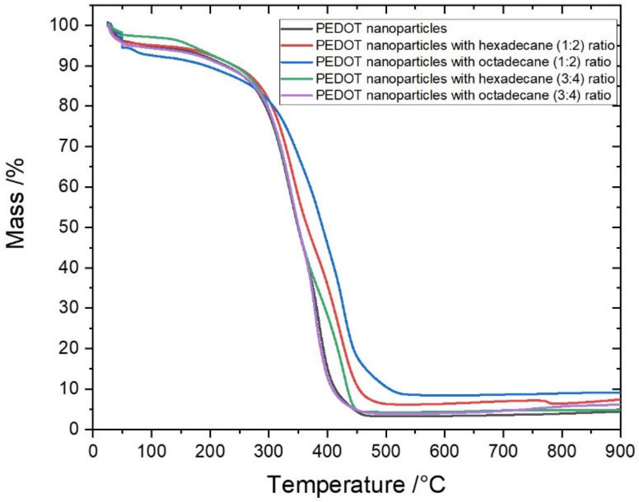

Figure S3. TGA curves of PEDOT nanoparticles prepared without and with PCM: no alkane (black), EDOT:hexadecane ratio of 2:1 (red), EDOT:octadecane ratio of 2:1 (blue), EDOT:hexadecane ratio of 4:3 (green), and EDOT:octadecane ratio of 4:3 (magenta).

5. X-ray Diffraction Patterns

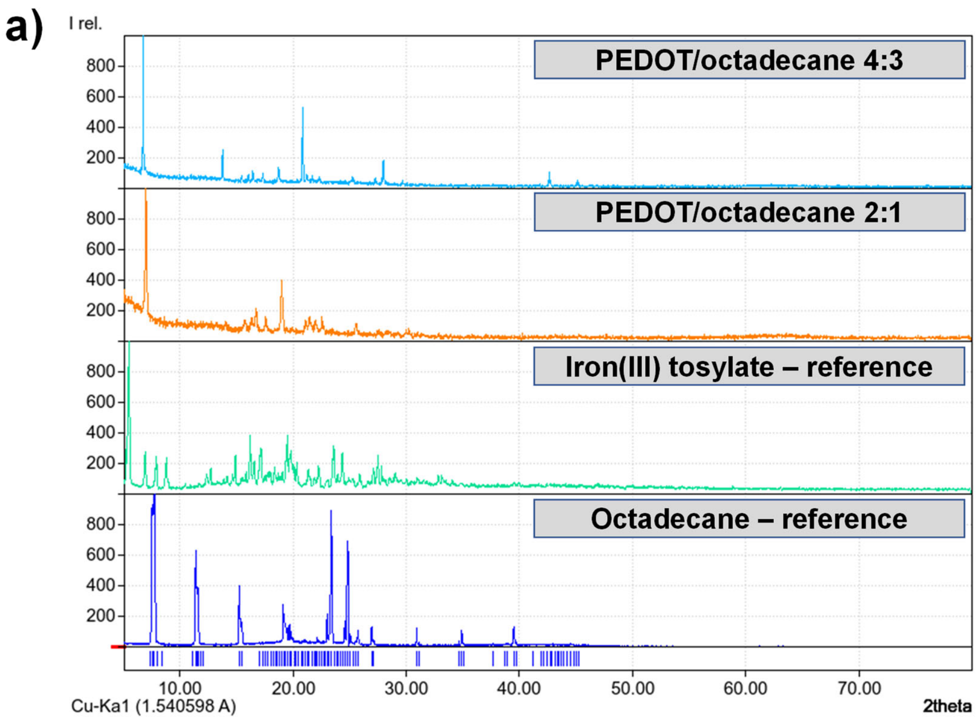

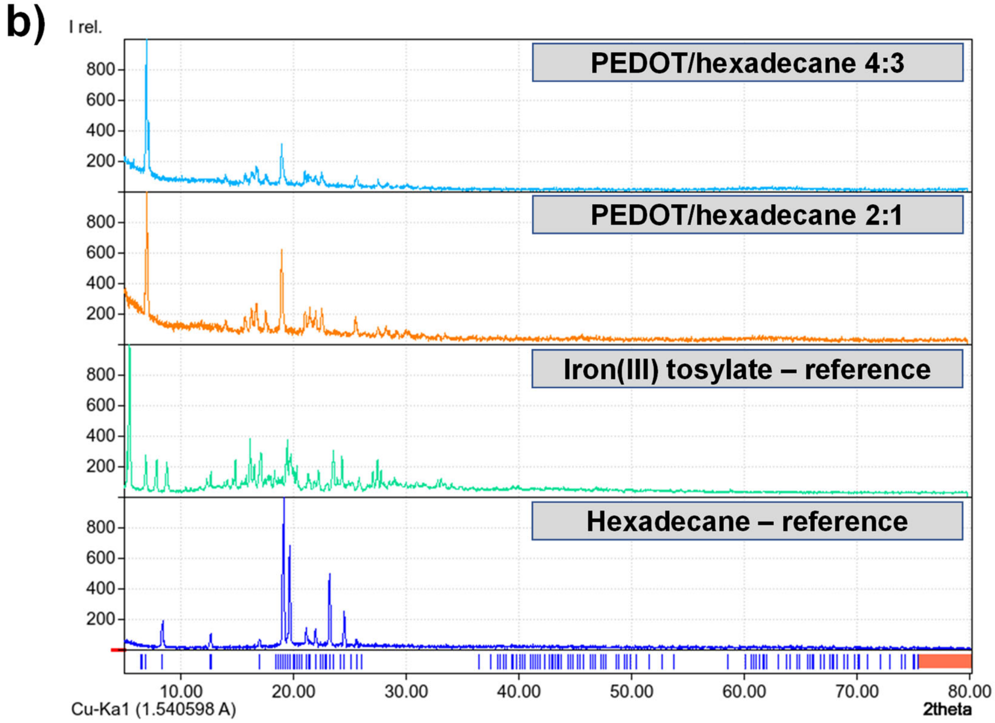

Figure S4. X-ray diffraction patterns of (a) PEDOT/octadecane and (b) PEDOT/hexadecane nanoparticles prepared at EDOT:PCM ratios of 2:1 and 4:3. Octadecane, hexadecane, and iron(III) tosylate are also included as references.

6. DSC Curves of PEDOT Nanoparticles

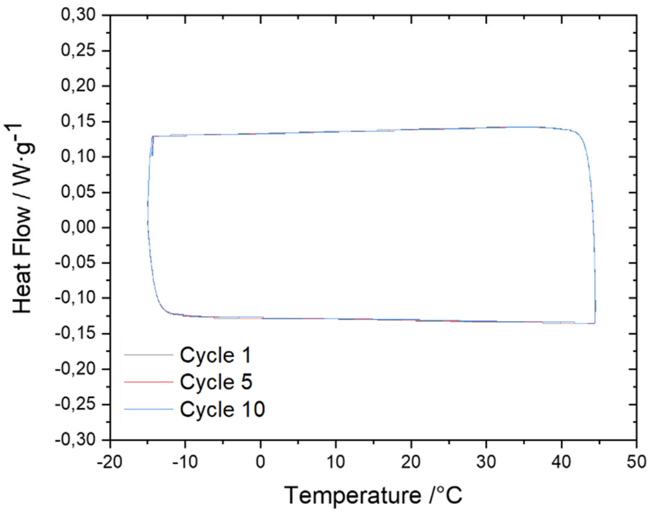

Figure S5. DSC curves of PEDOT nanocapsules without PCM.

7. System for Heat Transfer Measurement

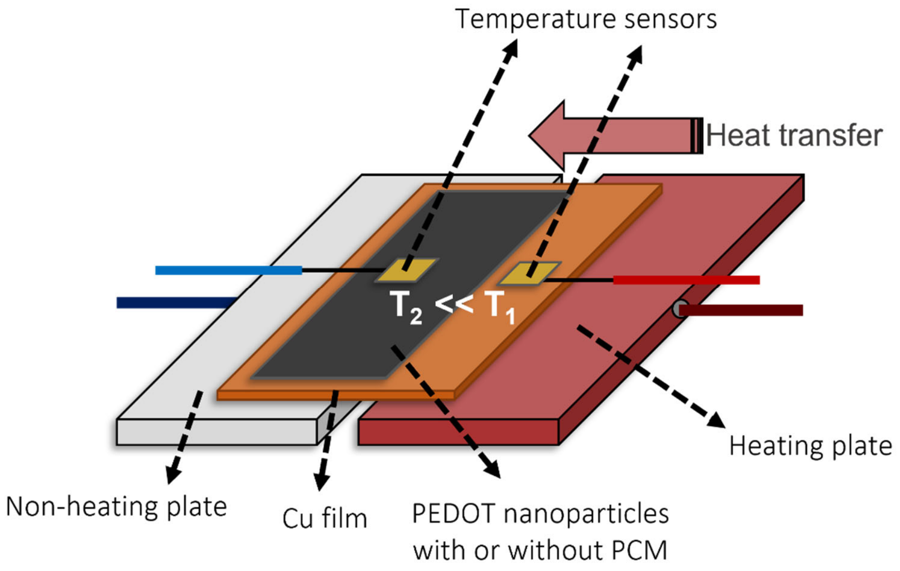

Figure S6. Representation of the heat transfer measurement system on the Cu film.
